# Detection of fibrotic remodeling of epicardial adipose tissue in patients with atrial fibrillation: Imaging approach based on histological observation

**DOI:** 10.1016/j.hroo.2021.05.006

**Published:** 2021-05-24

**Authors:** Yumi Ishii, Ichitaro Abe, Shintaro Kira, Taisuke Harada, Masayuki Takano, Takahiro Oniki, Hidekazu Kondo, Yasushi Teshima, Kunio Yufu, Takashi Shuto, Tomoyuki Wada, Mikiko Nakagawa, Tatsuo Shimada, Yoshiki Asayama, Shinji Miyamoto, Naohiko Takahashi

**Affiliations:** ∗ Department of Cardiology and Clinical Examination; † Department of Cardiovascular Surgery; ‡ Medical Education Center, and; ‖ Department of Radiology, Oita University Faculty of Medicine, Oita, Japan; § Oita Medical Technology School, College of Judo Therapy and Acupuncture-Moxibustion, Oita, Japan

**Keywords:** Atrial fibrillation, Computed tomography, Epicardial adipose tissue, Fat attenuation, Fibrosis, Inflammation

## Abstract

**Background:**

Fibrotic remodeling of epicardial adipose tissue (EAT) is crucial for proinflammatory atrial myocardial fibrosis, which leads to atrial fibrillation (AF).

**Objectives:**

We tested the hypothesis that the ratio of central to marginal adipocyte diameter in EAT represents its fibrotic remodeling. Based on a similar concept, we also tested whether the percent (%) change in EAT fat attenuation determined using computed tomographic (CT) images can detect this remodeling.

**Methods:**

Left atrial appendages were obtained from 76 consecutive AF patients during cardiovascular surgery. EAT in the central area (central EAT: C-EAT) and that adjacent to the atrial myocardium (Marginal EAT: M-EAT) were evaluated histologically. CT images for all of the 76 patients were also analyzed.

**Results:**

The adipocyte diameter was smaller, fibrotic remodeling of EAT (EAT fibrosis) was more severe, and infiltration of macrophages and myofibroblasts was more extensive in M-EAT than in C-EAT. EAT fibrosis was positively correlated with adipocyte diameter in C-EAT and negatively correlated in M-EAT, resulting in a positive correlation between EAT fibrosis and the ratio of central to marginal adipocyte diameter (C/M diameter ratio; r = 0.73, *P* < .01). The C/M diameter ratio was greater in patients with persistent AF than in those with paroxysmal AF. CT images demonstrated that the %change in EAT fat attenuation was positively correlated with EAT fibrosis.

**Conclusion:**

Our results suggest that the central-to-marginal adipocyte diameter ratio is tightly associated with fibrotic remodeling of EAT. In addition, the %change in EAT fat attenuation determined using CT imaging can detect remodeling noninvasively.

**Key Findings**

▪ The diameter of adipocytes was smaller, fibrotic remodeling of epicardial adipose tissue (EAT) was more severe, and infiltration of macrophages and myofibroblasts was more abundant in marginal EAT (M-EAT) compared with central EAT (C-EAT).
▪ EAT fibrosis was positively correlated with adipocyte diameter in C-EAT but negatively correlated with adipocyte diameter in M-EAT, resulting in a tight positive correlation between EAT fibrosis and the ratio of central to marginal adipocyte diameter (C/M diameter ratio).
▪ The percent change in EAT fat attenuation using computed tomography images was positively correlated with EAT fibrosis.
▪ EAT fibrosis, myocardial fibrosis, total collagen in myocardium, C/M diameter ratio, and percent change in EAT fat attenuation were greater in patients with persistent atrial fibrillation (AF) than in patients with paroxysmal AF.

## Introduction

The volume of epicardial adipose tissue (EAT) has been shown to be associated with atrial fibrillation (AF), independent of traditional risk factors.^1^ In this regard, Haemers and colleagues^2^ demonstrated that subepicardial fatty infiltration (fibro-fatty infiltration) in right atrial samples obtained during cardiovascular surgery contributes to structural remodeling, forming a substrate for AF. Using left atrial (LA) appendage (LAA) samples obtained from 59 consecutive AF patients during cardiovascular surgery, we recently reported that the severity of fibrotic remodeling of EAT was associated with LA myocardial fibrosis and the progression of AF.^3^ We also found that proinflammatory and profibrotic cytokines/chemokines, including interleukin (IL)-6, monocyte chemoattractant protein-1, and tumor necrosis factor (TNF)-α in peri-LA EAT were associated with atrial myocardial fibrosis.^3^^,^^4^ Commonly, expansion of adipose tissue mass is characterized by an increase in the size and/or number of adipocytes.^5^ In visceral adipose tissue (VAT), however, fibrosis has been reported to negatively correlate with adipocyte diameter.^6^ We therefore firstly tested the hypothesis that the ratio of central to marginal adipocyte diameter in EAT might represent fibrotic remodeling of EAT, a crucial state for promoting atrial myocardial fibrosis and forming a substrate for AF.

Antonopoulos and colleagues^7^ recently presented a method for detecting coronary inflammation by characterizing the changes in peri-coronary adipose tissue computed tomography (CT) attenuation. In a large cohort of patients undergoing cardiac surgery, they demonstrated that the average attenuation of adipose tissue is inversely correlated with the average adipocyte size, which is driven by intracellular lipid accumulation.^7^ Based on a similar concept, we tested the hypothesis that the percent (%) change in EAT fat attenuation determined using CT images can predict the fibrotic remodeling of EAT noninvasively.

## Methods

This study protocol was approved by the Ethics Committee of Oita University Hospital. This study was conducted in accordance with the guidelines proposed in the Declaration of Helsinki. Materials and methods are described in detail in the Supplemental Materials.

### Definition of central EAT and marginal EAT

**Figure 1:**
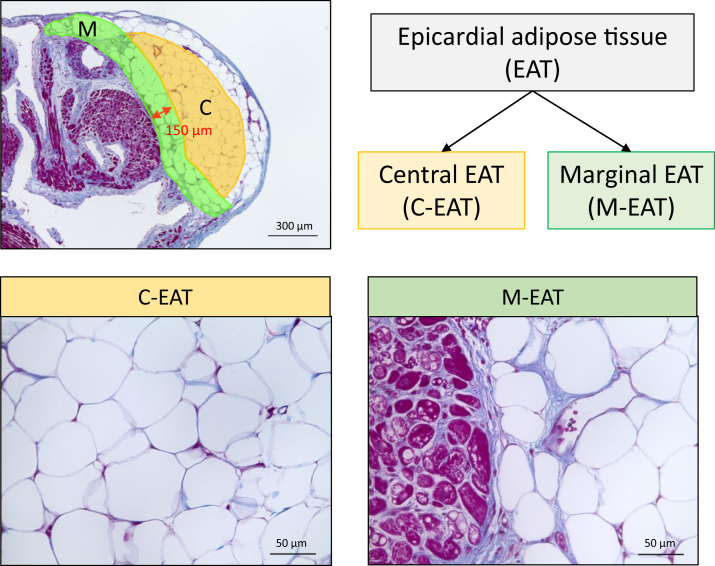
Definition of 2 types of epicardial adipose tissue (EAT). Central EAT (C-EAT) was defined as the central area of EAT that is not attached to either the myocardium or epicardium (*orange area*). Marginal EAT (M-EAT) was defined as the area within 150 μm from the myocardium, including the adipocytes in contact with the myocardium (*green area*).

To study the phenotypic differences between the adipose tissue in contact with the atrial myocardium and the adipose tissue separated from the atrial myocardium, we divided the EAT into the following 2 areas: (1) central EAT (C-EAT); and (2) marginal EAT (M-EAT) (Figure 1). C-EAT was defined as the central area of EAT that is not attached to either the myocardium or epicardium. M-EAT was defined as the area within 150 μm from the myocardium, including the adipocytes in contact with the myocardium.

### Quantitative real-time polymerase chain reaction

Quantitative real-time polymerase chain reaction was performed as previously described.^8^ Primer sequences and conditions are listed in Supplemental Table S1.

### Statistical analyses

Continuous data were evaluated for normality using the Shapiro-Wilk test. Normally distributed continuous data are expressed as the mean ± standard deviation. The Student *t* test was used for comparison between groups if the data were normally distributed. The Wilcoxon test was used for nonparametric tests. Correlations between continuous variables were assessed by bivariate analysis, and the Pearson coefficient (r) was estimated.

All statistical tests were performed with JMP v.14 software (SAS, Cary, NC). A value of *P* < .05 was considered to be statistically significant.

## Results

### Patient characteristics

Clinical characteristics of the 76 patients are summarized in Table 1. The mean age was 71.9 ± 8.2 years. Thirty-six patients (47%) were female. The mean CHADS_2_ and CHA_2_DS_2_-VASc scores for all patients were 2.6 ± 1.1 and 4.0 ± 1.4, respectively. Twenty-eight patients (37%) had paroxysmal AF (PAF group), while 48 patients (63%) had persistent AF (PeAF group). EAT volume, assessed by CT imaging, was greater in the PeAF group than in the PAF group (99 ± 51 vs 71 ± 28 mL, *P* < .05).

**Table 1:** Patient characteristics

	All patients (n = 76)	Paroxysmal AF (n = 28)	Persistent AF (n = 48)	*P* value
Age (years)	71.9 ± 8.2	69.9 ± 10.2	73.2 ± 6.7	.09
Sex				
Male	40 (53)	12 (43)	28 (58)	.19
Female	36 (47)	16 (57)	20 (42)	.19
BMI (kg/m^2^)	23.2 ± 3.6	22.6 ± 2.7	23.6 ± 4.0	.24
History of present and past illness				
Hypertension	39 (51)	14 (50)	25 (52)	.86
Diabetes mellitus	18 (24)	4 (14)	14 (29)	.14
Dyslipidemia	30 (39)	12 (43)	18 (38)	.64
Coronary artery disease	17 (22)	7 (25)	10 (21)	.67
Cerebral infarction	14 (18)	6 (21)	8 (17)	.61
Sleep apnea	1 (1)	0 (0)	1 (1)	.44
Smoking	28 (37)	11 (39)	17 (35)	.74
Alcohol use	22 (29)	4 (14)	18 (38)	<.05
CHADS_2_ score	2.6 ± 1.1	2.5 ± 1.1	2.6 ± 1.1	.50
CHA_2_DS_2_-VASc score	4.0 ± 1.4	4.0 ± 1.3	4.1 ± 1.5	.85
Surgical procedure				
Valve replacement/repair	51 (67)	21 (75)	30 (63)	.26
Aorta replacement	6 (8)	0 (0)	6 (13)	.05
CABG	1 (1)	0 (0)	1 (2)	.44
Combined (CABG and valve)	14 (18)	7 (25)	7 (15)	.26
Combined (aorta and valve)	3 (4)	0 (0)	3 (6)	.17
Combined (ASD closure and valve)	1 (1)	0 (0)	1 (2)	.44
BUN (mg/dL)	25 ± 13	23 ± 14	25 ± 12	.39
Cr (mg/dL)	1.4 ± 1.8	1.8 ± 2.8	1.2 ± 0.6	.13
BNP (pg/mL)	358 ± 435	430 ± 500	311 ± 388	.31
eGFR (mL/min/1.73 m^2^)	51 ± 20	53 ± 22	50 ± 19	.45
LAD (mm)	52 ± 10	47 ± 8	54 ± 11	<.01
LVDd (mm)	53 ± 9	52 ± 9	53 ± 9	.45
EF (%)	59 ± 12	62 ± 13	58 ± 13	.13
E/e'	22 ± 13	25 ± 13	20 ± 12	.11
AR II°-III°	19 (25)	5 (18)	14 (29)	.27
AS moderate-severe	18 (24)	8 (29)	10 (21)	.44
MR II°-III°	47 (62)	17 (61)	30 (63)	.88
MS moderate-severe	11 (14)	6 (21)	5 (10)	.19
EAT volume (mL)	89 ± 46	71 ± 28	99 ± 51	<.05
EAT volume corrected by BMI	3.8 ± 1.6	3.1 ± 1.1	4.1 ± 1.8	<.01

Data are given as mean ± SD or n (%).

AF = atrial fibrillation; AR = aortic regurgitation; AS = aortic stenosis; ASD = atrial septal defect; BMI = body mass index; BNP = brain natriuretic peptide; BUN = blood urea nitrogen; CABG = coronary artery bypass graft; Cr = creatinine; E/e = the ratio of the peak early mitral inflow velocity (E) over the early diastolic mitral annular velocity (e′); EAT = epicardial adipose tissue; EF = ejection fraction; eGFR = estimated glomerular filtration rate; LAD = left atrial diameter; LVDd = left ventricular end-diastolic diameter; MR = mitral regurgitation; MS = mitral stenosis.

### Fibrotic remodeling of EAT (EAT fibrosis) and adipocyte diameters in C-EAT and M-EAT

**Figure 2:**
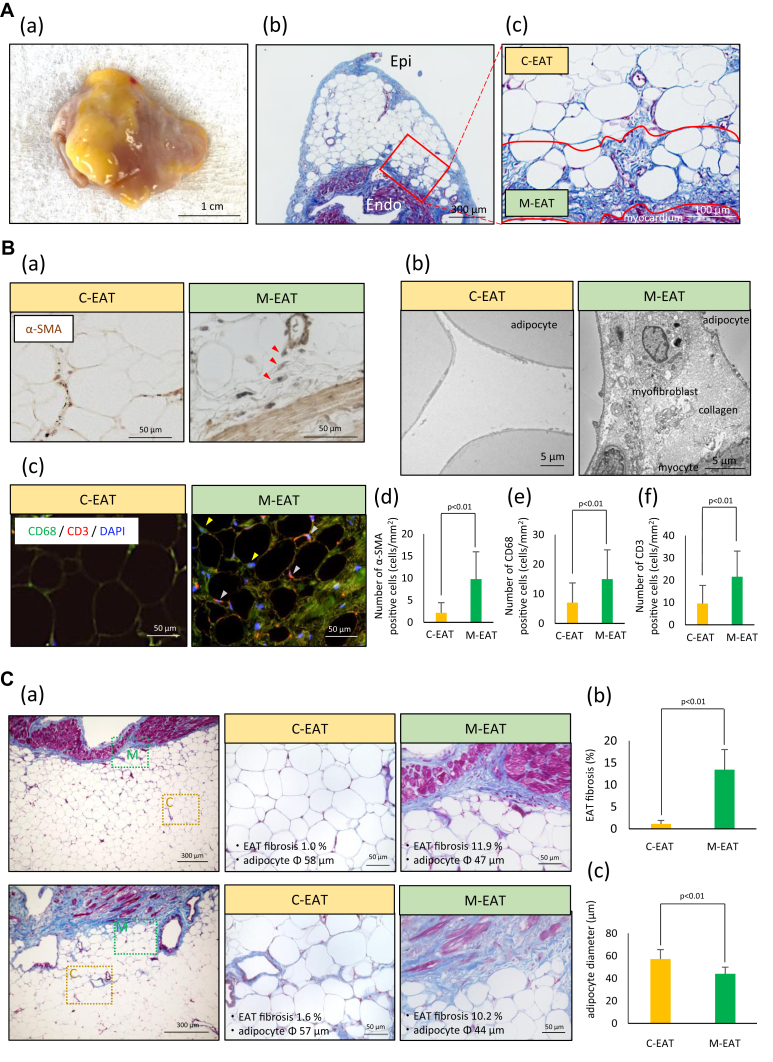
Fibrotic remodeling of epicardial adipose tissue (EAT) and adipocyte diameter. **A:** (a) Representative photomicrograph of excised left atrial appendage, and (b)(c) sections with Masson’s trichrome staining. **B:** Representative photomicrographs of (a) immunostaining for α-SMA (*red arrows*), (b) transmission electron microscope imaging, (c) immunostaining for CD68 (*yellow arrows*) and CD3 (*silver arrows*). CD68, green; CD3, red and DAPI, blue. (d)–(f) Corresponding quantitative analysis of (a)–(c). **C:** (a) Representative cases and (b)(c) corresponding quantitative analysis. C-EAT = central EAT; Endo = endocardium; Epi = epicardium; M-EAT = marginal EAT.

Macroscopically, excised LAA samples were invariably surrounded by EAT (Figure 2A(a), Supplemental Figure S1). Fibrotic remodeling of EAT was observed at the marginal area (M-EAT) but not in the central area (C-EAT) (Figure 2A(b)(c), Supplemental Figure S2). Masson’s trichrome staining revealed that atrial myocardial fibrosis correlated with EAT fibrosis (r = 0.51, *P* < .01) and that EAT fibrosis was greater in the PeAF group than in the PAF group (11.0% ± 3.6% vs 14.9% ± 4.5%, *P* < .01) (Supplemental Figure S3A and S3B). In M-EAT, abundant infiltration by α-smooth muscle actin (SMA)-positive cells was observed (Figure 2B(a)). The α-SMA-positive cells were morphologically identified as myofibroblasts by transmission electron microscopy (Figure 2B(b)), and the infiltration of CD68-positive and CD3-positive cells were abundantly observed in M-EAT (Figure 2B(c)). In all 76 cases, the number of α-SMA-positive cells was greater in M-EAT than in C-EAT (9.8 ± 6.2 cells/mm^2^ vs 2.2 ± 2.3 cells/mm^2^, *P* < .01) (Figure 2B(d)). Similarly, the number of CD68-positive and CD3-positive cells was greater in M-EAT than in C-EAT (15.0 ± 9.9 vs 7.0 ± 6.7 cells/mm^2^ and 21.6 ± 11.5 vs 9.5 ± 8.1 cells/mm^2^, respectively, both *P* < .01) (Figure 2B(e)(f)). Representative Masson’s trichrome images of sections from 2 individual patients in which the area of fibrotic remodeling of EAT (EAT fibrosis) and the adipocyte diameter were measured are shown in Figure 2C(a). In these 2 cases, fibrosis was rare in C-EAT but abundant in M-EAT. Adipocyte diameter was larger in C-EAT than in M-EAT. Quantitative analyses of all 76 cases revealed less fibrosis in C-EAT than in M-EAT (1.1% ± 0.8% vs 13.4% ± 4.6%, *P* < .01) (Figure 2C(b)). Adipocyte diameter was smaller in M-EAT than in C-EAT (44 ± 5.9 μm vs 57 ± 8.4 μm, *P* < .01) (Figure 2C(c)).

### Correlation of adipocyte diameter with body mass index and EAT volume

**Figure 3:**
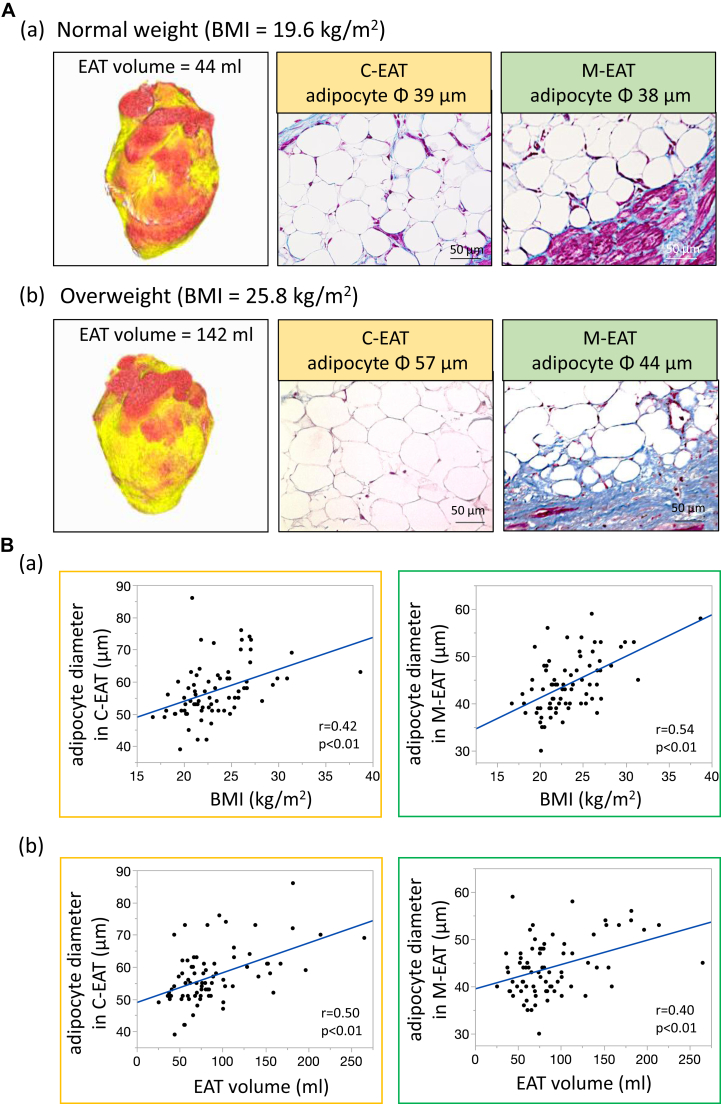
Relationship of adipocyte diameter with body mass index (BMI) and epicardial adipose tissue (EAT) volume. **A:** (a)(b) Representative examples; **B:** (a)(b) correlation of adipocyte diameter with BMI and EAT volume. C-EAT = central EAT; M-EAT = marginal EAT.

CT images and Masson’s trichrome staining of samples from 2 representative patients (normal weight and overweight) are shown in Figure 3A(a) and 3A(b). In all 76 cases, adipocyte diameters in both C-EAT and M-EAT were positively correlated with body mass index (BMI) (both *P* < .01) (Figure 3B(a)). Similarly, adipocyte diameters in both C-EAT and M-EAT were positively correlated with EAT volume (both *P* < .01) (Figure 3B(b)).

### Gene expression profile of C-EAT and M-EAT by microarray analysis

**Figure 4:**
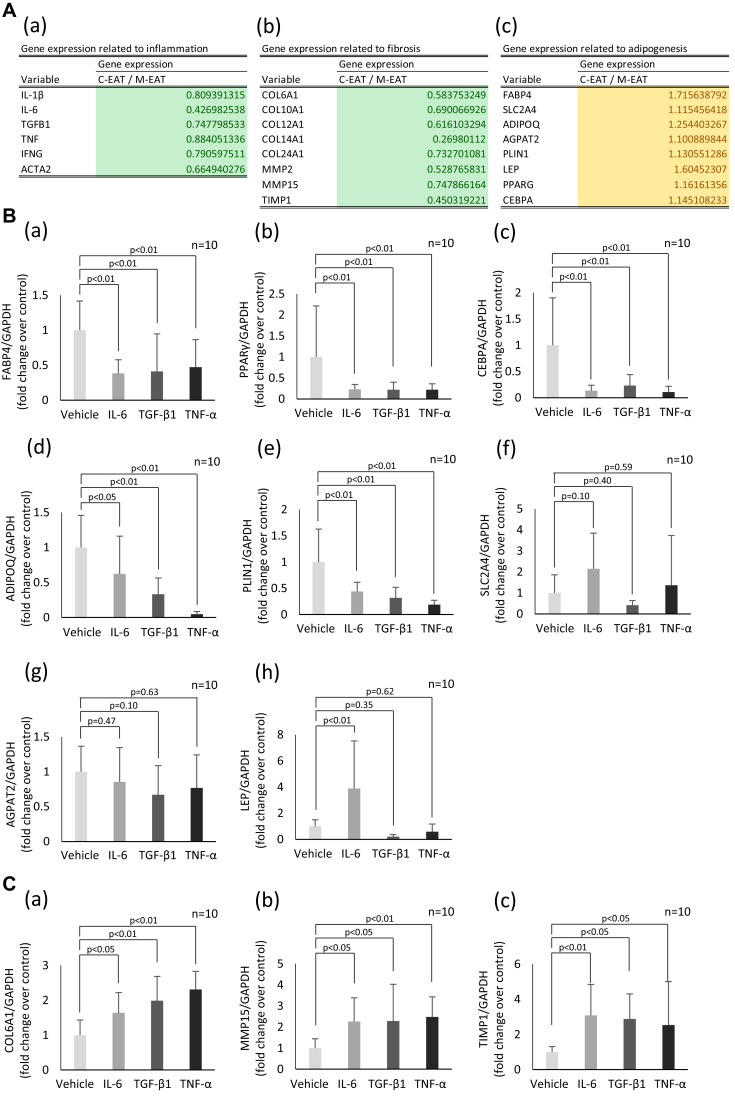
Microarray analyses and effects of cytokines on expression levels of adipogenesis-related mRNAs. **A:** (a)–(c) Downregulation (*green*) and upregulation (*yellow*) of inflammation-, fibrosis-, and adipogenesis-related genes in central epicardial adipose tissue (C-EAT) compared with marginal EAT (M-EAT). **B:** (a)–(h) Gene expression related to adipogenesis in EAT treated with IL-6 (25 ng/mL), TGF-β1 (1 ng/mL), and TNF-α (5 ng/mL).

To test the hypothesis that specific cytokines in M-EAT may inhibit the normal development of preadipocytes, resulting in smaller adipocytes in M-EAT, microarray analysis of 2 distinct adipocyte clusters obtained from C-EAT and M-EAT was carried out using adipocytes from 3 randomly selected patients. The EAT areas isolated and evaluated are illustrated in Supplemental Figure S4, and the results of the microarray analysis are shown in Figure 4A(a–c) and Supplemental Table S2. In these 3 cases, 871 genes were upregulated and 1741 genes were downregulated in C-EAT compared with M-EAT (Supplemental Table S2). Genes associated with inflammation, including IL-6, transforming growth factor (TGF)-β, and TNF, were downregulated in C-EAT (Figure 4A(a)). Genes associated with fibrosis, including *COL*, *MMP*, and *TIMP*, were also downregulated in C-EAT (Figure 4A(b)). On the other hand, genes associated with adipogenesis, including *FABP4*, *PPARG*, and *CEBPA*, were upregulated in C-EAT (Figure 4A(c)).

### Effects of proinflammatory cytokines on expression of adipogenic and profibrotic genes in cultured human EAT

EAT was isolated from LAA of 6 randomly selected patients and cultured with the proinflammatory cytokines that were shown to be downregulated in C-EAT (upregulated in M-EAT) (Figure 4A(a)). Expression levels of mRNAs associated with adipogenesis after 24 hours of exposure to the cytokines are shown in Figure 4B. The mRNA expression levels of *FABP4*, *PPARγ*, and *CEBPA* were reduced in EAT incubated with IL-6, TGF-β1, and TNF-α compared with EAT incubated with vehicle (all *P* < .01) (Figure 4B(a–c)). The mRNA expression levels of *ADIPOQ* and *PLIN1* were suppressed by IL-6, TGF-β1, and TNF-α (Figure 4B(d) and 4B(e)). The mRNA expression levels of *SLC2A4* and *AGPAT2* were not significantly affected by IL-6, TGF-β1, and TNF-α (Figure 4B(f) and 4B(g)). In contrast, the mRNA expression levels of *LEP* were upregulated by IL-6, but not by TGF-β1 and TNF-α (Figure 4B(h)). The mRNA expression levels of *COL6A1*, *MMP15*, and *TIMP1* were upregulated by IL-6, TGF-β1, and TNF-α (Figure 4C(a–c)).

### Ratio of adipocyte diameter in C-EAT to that in M-EAT and EAT fibrosis

**Figure 5:**
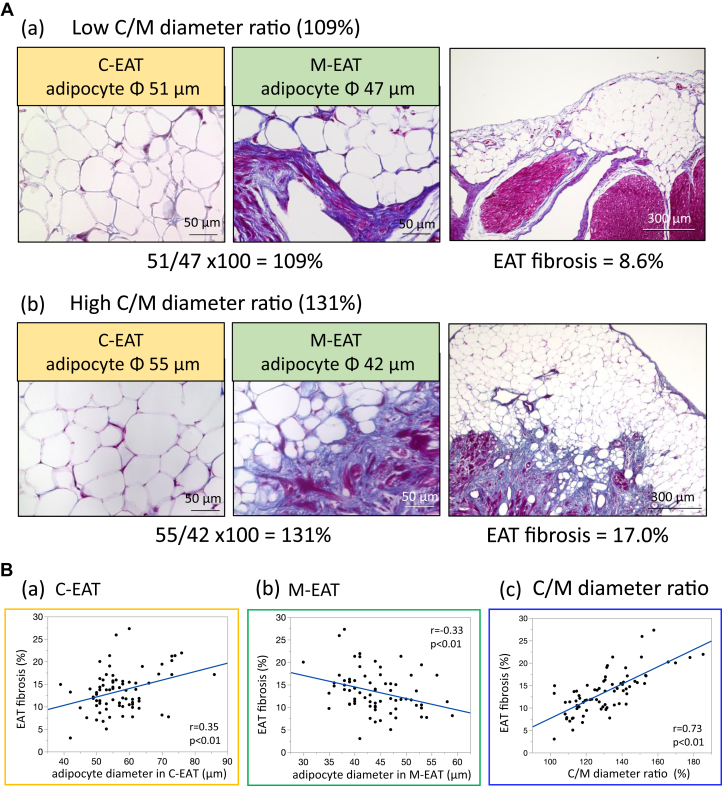
Central-to-marginal (C/M) ratio of adipocyte diameter. **A:** (a)(b) Representative examples. **B:** Correlation of epicardial adipose tissue (EAT) fibrosis with (a)(b) adipocyte diameter and (c) C/M diameter ratio (ratio of adipocyte diameter in central EAT [C-EAT] to that in marginal EAT [M-EAT]).

In samples from 1 patient, the adipocyte diameter in C-EAT (51 μm) was almost equal to that in M-EAT (47 μm) (Figure 5A(a)), resulting in a ratio of adipocyte diameter in C-EAT to that in M-EAT (C/M diameter ratio) of 109%. In this case, EAT fibrosis was 8.6%. In samples from another patient, the adipocyte diameter in C-EAT (55 μm) was clearly larger than that in M-EAT (42 μm), resulting in a C/M diameter ratio of 131% (Figure 5A(b)). In this case, EAT fibrosis was 17.0%. Quantitative analyses using all 76 cases demonstrated that EAT fibrosis was positively correlated with adipocyte diameter in C-EAT (r = 0.35, *P* < .01) (Figure 5B(a)). EAT fibrosis was, however, negatively correlated with adipocyte diameter in M-EAT (r = -0.33, *P* < .01) (Figure 5B(b)). EAT fibrosis thus showed a tight positive correlation with the C/M diameter ratio of adipocytes (r = 0.73, *P* < .01) (Figure 5B(c)).

### C/M diameter ratio and atrial myocardial fibrosis

**Figure 6:**
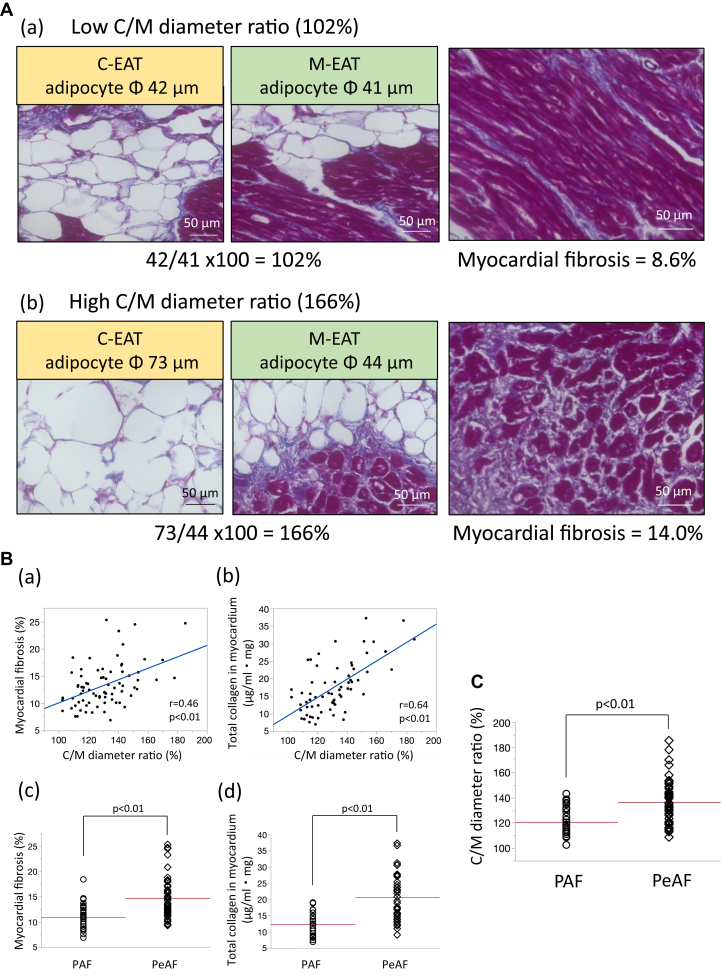
Central-to-marginal (C/M) diameter ratio and myocardial fibrosis. **A:** (a)(b) Representative cases. **B:** Correlation of C/M diameter ratio with (a) myocardial fibrosis and (b) total collagen in myocardium. (c) Myocardial fibrosis, (d) total collagen in myocardium, and **C:** C/M diameter ratio were higher in patients with persistent atrial fibrillation (PeAF) than paroxysmal atrial fibrillation (PAF).

Data for the representative patient with comparable adipocyte diameters in C-EAT and M-EAT and less atrial myocardial fibrosis are shown in Figure 6A(a) and data for the representative patient with larger adipocyte diameters in C-EAT than in M-EAT and more atrial myocardial fibrosis are shown in Figure 6A(b). Quantitative analyses using all 76 cases showed that atrial myocardial fibrosis was positively correlated with C/M diameter ratio (r = 0.46, *P* < .01) (Figure 6B(a)). Total collagen in atrial myocardium also showed a positive correlation with C/M diameter ratio (r = 0.64, *P* < .01) (Figure 6B(b)). Both atrial myocardial fibrosis and total collagen in the atrial myocardium were greater in the PeAF group than in the PAF group (14.9% ± 4.5% vs 11.0% ± 3.6% and 20.6 ± 7.1 μg/mL･mg vs 12.3 ± 3.8 μg/mL･mg, respectively, both *P* < .01) (Figure 6B(c) and 6B(d)). The C/M diameter ratio was greater in the PeAF group than in the PAF group (136.4% ± 17.5% vs 120.8% ± 12.1%, *P* < .01) (Figure 6C).

### Association of C/M diameter ratio of adipocytes with cytokines/chemokines in EAT

The C/M diameter ratio was positively correlated with the amounts of the following proteins: IL-1b, IL-2, IL-4, IL-6, IL-7, IL-9, IL-10, IL-12, IL-13, IL-17, eotaxin, granulocyte-colony stimulating factor, granulocyte macrophage-colony stimulating factor, monocyte chemoattractant protein-1, macrophage inflammatory protein-1b, platelet-derived growth factor-bb, RANTES, TNF-α, vascular endothelial growth factor, matrix metalloproteinase (MMP)2, MMP9, and angiopoietin-like protein2 (Supplemental Table S3).

### CT imaging to determine %change in EAT fat attenuation

**Figure 7:**
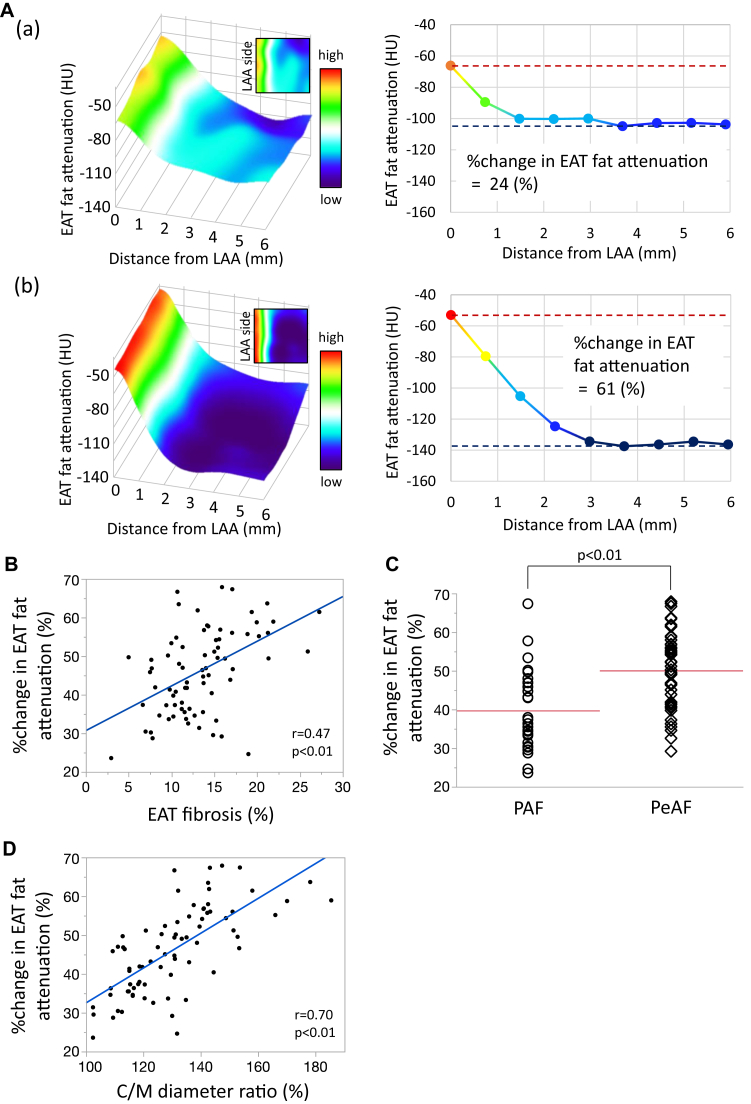
The percent change (%change) in epicardial adipose tissue (EAT) fat attenuation by computed tomography imaging. **A:** Representative heat map of EAT fat attenuation in mild (a) and severe fibrotic remodeling of EAT cases (b). **B:** Correlation between the %change in EAT fat attenuation and the EAT fibrosis. **C:** Comparison of the %change in EAT fat attenuation between paroxysmal atrial fibrillation (PAF) and persistent atrial fibrillation (PeAF). **D:** Correlation between the %change in EAT fat attenuation and the central-to-marginal (C/M) diameter ratio.

The representative EAT fat attenuation using CT images is shown in Supplemental Figure S5 and Figure 7A(a) and 7A(b). In a patient with mild fibrotic remodeling of EAT (EAT fibrosis = 7.6%), the %change in EAT fat attenuation was gradual and calculated to be 24% (Figure 7A(a)). In contrast, in a patient with severely fibrotic remodeling of EAT (EAT fibrosis = 27.3%), the %change in EAT fat attenuation was large and calculated to be 61% (Figure 7A(b)). The %change in EAT fat attenuation in all 76 patients was positively correlated with EAT fibrosis (r = 0.47, *P* < .01) (Figure 7B). The %change in EAT fat attenuation was greater in the PeAF group than in the PAF group (50.1% ± 9.8% vs 39.7% ± 10.4%, *P* < .01) (Figure 7C). The %change in EAT fat attenuation was positively correlated with the histologically assessed C/M diameter ratio (r = 0.70, *P* < .01) (Figure 7D).

### Influence of BMI and left atrial size

Myocardial fibrosis, total collagen in myocardium, C/M diameter ratio, and %change in EAT fat attenuation did not correlate with BMI. These 4 parameters remained significant predictors of PeAF after correction with respect to BMI. On the other hand, myocardial fibrosis (r = 0.33, *P* < .01), total collagen in myocardium (r = 0.30, *P* < .05), and C/M diameter ratio (r = 0.24, *P* < .05) all correlated with LAD. Because LAD itself was able to predict PeAF, the ability of C/M diameter ratio and %change in EAT fat attenuation to predict PeAF was not significant after correction with respect to LAD.

## Discussion

### Major findings

The main findings of the present study are as follows. First, the diameter of adipocytes was smaller, fibrotic remodeling of EAT was more severe, and infiltration of macrophages and myofibroblasts was more abundant in M-EAT compared with C-EAT. Second, EAT fibrosis was positively correlated with adipocyte diameter in C-EAT but negatively correlated with adipocyte diameter in M-EAT, resulting in a tight positive correlation between EAT fibrosis and the ratio of central to marginal adipocyte diameter (C/M diameter ratio). Third, the %change in EAT fat attenuation using CT images was positively correlated with EAT fibrosis. Finally, EAT fibrosis, myocardial fibrosis, total collagen in myocardium, C/M diameter ratio, and %change in EAT fat attenuation were greater in patients with PeAF than in patients with PAF.

In the histological study, the EAT that randomly attached to the tip of the LAA was analyzed. On the other hand, in the CT imaging study, a cross-sectional EAT close to the LAA was analyzed. This suggests that we did not evaluate exactly the same EAT between the histological and the CT imaging study. Nevertheless, this is the first study to demonstrate that qualitative assessment of EAT using CT imaging may detect fibrotic remodeling of EAT, based on the histological observations in the same subjects.

### Large-sized adipocytes in C-EAT and small-sized adipocytes in M-EAT

In agreement with a previous report,^5^ we found that adipocyte diameters in both C-EAT and M-EAT were positively correlated with BMI and EAT volume (Figure 3B). The expansion of adipocytes leads to activation of macrophages and a heightened inflammatory state.^9^^,^^10^ This has been suggested to result in hypoxic adipocytes, which initiate a vicious cycle of local proinflammatory activation.^11^ In the present study, the majority of EAT was present as C-EAT. Because EAT fibrosis was positively correlated with adipocyte diameter in C-EAT (Figure 5B(a)), it is conceivable that large-sized adipocytes in C-EAT are associated with fibrotic remodeling of EAT. On the other hand, Divoux and colleagues^6^ found that fibrosis of omental white adipose tissue, a type of VAT, was negatively correlated with the diameters of omental adipocytes in surgical biopsies of subcutaneous white adipose tissue and omental adipose tissue taken from 65 obese subjects undergoing bariatric surgery. Interestingly, they concluded that fibrosis may play a role limiting adipocyte hypertrophy in omental adipose tissue.^6^ EAT is an ectopic adipose tissue and has been shown to have a phenotype closer to that of VAT, with smaller adipocytes.^7^ The findings by Divoux and colleagues^6^ in omental adipose tissue are, in any case, consistent with our own observation that EAT fibrosis was negatively correlated with adipocyte diameter in M-EAT. We would like to emphasize the tight positive correlation between EAT fibrosis and the C/M adipocyte diameter ratio (Figure 5B(c)), which suggested that both the larger adipocytes in C-EAT and the smaller adipocytes in M-EAT were closely associated with the severity of fibrotic remodeling of EAT.

### Mechanisms for reduction in size of adipocytes in M-EAT

In agreement with Divoux and colleagues,^6^ a recent review article^12^ concluded that visceral fibrosis may play a role in limiting adipocyte expansion, thus acting as an adaptive mechanism to reduce the negative effect of adipocyte hypertrophy. Such a mechanism could explain the reduced size of adipocytes in M-EAT, as shown in Figure 2. In this regard, Lee and colleagues^13^ recently demonstrated that glucocorticoids restrain cell-autonomous TGF-β signaling in adipose stem cells and thus facilitate adipogenesis and healthy remodeling in abdominal subcutaneous adipose tissue and that these processes are impaired in omental adipose tissue. In the case of adipogenesis, we previously showed that the concentrations of IL-6 and TNF-α in EAT were associated with atrial myocardial fibrosis.^3^ It is noteworthy that IL-6 and TNF-α have been reported to inhibit the normal development of preadipocytes and to promote a proinflammatory phenotype.^14^ TGF-β1 also reportedly caused differentiated adipocytes to revert to a state characteristic of preadipocytes.^15^ In our microarray analysis, mRNAs corresponding to IL-6, TNF-α, and TGF-β1 were more highly expressed in M-EAT than in C-EAT (Figure 4A(a)). Incubation of cultured EAT with IL-6, TNF-α, and TGF-β1 also suppressed expression of mRNAs for *FABP4*, *CEBPA*, and *PPARγ* (Figure 4B(a–c)), all of which are known to promote adipogenesis.^13^^,^^16^ Based on these findings, we suggested that IL-6, TNF-α, and TGF-β1 contributed to the smaller size of adipocytes in M-EAT, at least in part, by suppressing adipogenesis.

We have previously demonstrated a correlation between the protein content of total collagen in atrial myocardium and the protein content of cytokines/chemokines in EAT.^3^ In a previous study,^3^ the protein content of total collagen showed the strongest correlation with TNF-α in EAT (r = 0.452, *P* = .0006). Therefore, we hypothesized that the C/M diameter ratio correlates with the protein content of these cytokines/chemokines in EAT. As expected, the C/M diameter ratio showed the strongest correlation with TNF-α in EAT (r = 0.579, *P* < .0001; Supplemental Table S3).

### %change in EAT fat attenuation in CT imaging

CT images of adipose tissue with larger adipocytes show lower attenuation because of the increased oil droplet size.^17^ Poorly differentiated smaller adipocytes show higher attenuation in CT images.^18^^,^^19^ Tissue inflammation and subsequent fibrosis can also lead to increase the tissue attenuation in CT scans.^20^ In a large cohort of patients undergoing cardiac surgery, Antonopoulos and colleagues^7^ demonstrated that the average attenuation of adipose tissue is inversely correlated with the expression of adipogenic genes and average adipocyte size, which is driven by intracellular lipid accumulation. More recently, Goeller and colleagues^21^ showed that the attenuation of peri-coronary adipose tissue measured from routine CT angiography is related to the progression of noncalcified plaque. This concept can also be applied to a study by Ciuffo and colleagues,^22^ who used mean CT attenuation in a standard 4-chamber view to measure the quality and quantity of peri-LA fat tissue by area (mm^2^) in 143 consecutive patients with drug-refractory AF referred for the first catheter AF ablation. Because LA fat attenuation correlated with peri-LA fat volume and was associated with AF recurrence, they concluded that peri-LA fat attenuation assessments can improve AF ablation outcomes by refining patient selection. The present study may be more valuable because it compared EAT fat attenuation using CT imaging with histological findings in each patient. In fact, the %change in EAT fat attenuation in CT imaging was well correlated with the histologically assessed C/M diameter ratio (Figure 7D).

### Clinical implications

Our results suggested that fibrotic EAT remodeling is crucial to promote atrial myocardial fibrosis and AF and can be detected by CT imaging. There are many patients who undergo coronary artery CT angiography examinations. The evaluation of EAT fat attenuation in these individuals may identify subjects who are at a high risk for developing new AF.

### Limitations

Several limitations existed in this study. First, the present study did not include a control group without AF. Second, 3 patients were randomly selected for the microarray analysis. The clinical characteristics of these 3 patients were generally similar to those of all 76 patients studied (data not shown). However, the influence of AF type and risk factors on gene expression in C-EAT in comparison to M-EAT remains unclear owing to the limited number of the analyzed patients. Third, we retrospectively analyzed CT scans that were performed before cardiovascular surgeries. To improve spatial resolution and reduce the radiation dose, the field of view was manually corrected by radiology technologists according to the patient physique (field of view, median 365 mm [interquartile range, 350–387.5 mm]). Therefore, there were individual differences in pixel size (pixel size, median 0.71 mm [0.68–0.76 mm]). These differences slightly reduced the accuracy in calculating the %change in EAT fat attenuation.

## Conclusion

Our results suggest that the central-to-marginal adipocyte diameter ratio was tightly associated with fibrotic remodeling of EAT and severity of AF. The results also suggest that the %change in EAT fat attenuation using a CT image approach can detect the fibrotic remodeling of EAT noninvasively.
