# Supplementary material for: Detection of fibrotic remodeling of epicardial adipose tissue in patients with atrial fibrillation: Imaging approach based on histological observation
Source: Heart Rhythm O2. 2021 May 24;2(4):311–23. doi: 10.1016/j.hroo.2021.05.006 (PMC8369308; doi:10.1016/j.hroo.2021.05.006)
Supplement: Supplemental Materials [file mmc1.docx]

**Supplemental materials**

**TITLE: Detection of Fibrotic Remodeling of Epicardial Adipose Tissue in Patients with Atrial Fibrillation: Imaging Approach Based on Histological Observation**

**AUTHORS:** Yumi Ishii, Ichitaro Abe, Shintaro Kira, Taisuke Harada, Masayuki Takano, Takahiro Oniki, Hidekazu Kondo, Yasushi Teshima, Kunio Yufu, Takashi Shuto, MD, PhD, Tomoyuki Wada, Mikiko Nakagawa, Tatsuo Shimada, Yoshiki Asayama, Shinji Miyamoto, Naohiko Takahashi

**Supplemental Methods**

This study protocol was approved by the Ethics Committee of Oita University Hospital (Approval number: 794). Written informed consent was obtained from all patients. This study was conducted in accordance with the guidelines proposed in the Declaration of Helsinki and registered at the University Hospital Medical Information Network (UMIN) Clinical Trials Registry (UMIN000026132).

**Study populations**

We enrolled 76 consecutive atrial fibrillation (AF) patients (36 females; age 71.9 ± 8.2 years) who underwent left atrial appendage (LAA) excision during cardiovascular surgery from January 2015 to April 2020. All patients were diagnosed with paroxysmal or persistent AF. The AF burden was classified according to American Heart Association/American College of Cardiology/Heart Rhythm Society guidelines ^1, 2^. All patients were evaluated by blood sampling test, echocardiography and thoracic computed tomography (CT) before surgery. LAA samples were collected during operation. They were rinsed with phosphate-buffered saline (PBS) and immediately preserved in the optimal storage environment (**Figure S1**).

**Histological study of LAA samples**

The LAA samples of 76 patients were fixed in 4% paraformaldehyde, embedded in paraffin, and cut into 5-μm sections for histological analysis. To analyze the association between epicardial adipose tissue (EAT) and the atrial myocardium, two sections were prepared per specimen. One section was created in a plane parallel to the trabeculae carneae and the other section was created in a plane vertical to trabeculae carneae as previously described ^3^. After deparaffinization, serial sections were stained with hematoxylin and eosin (HE) or Masson’s trichrome (MT). Images were acquired and digitized on a BIOLEVO BZ-9000 epifluorescence microscope (Keyence, Osaka, Japan).

As shown in **Figure S2A**, fibrosis area was measured using previous methods ^3^ in each area of the section, Central-EAT (C-EAT), Marginal-EAT (M-EAT), and Myocardium. The adipocyte diameter was measured in two distinct areas (C-EAT and M-EAT). First, the ROI was drawn manually according to the definition for C-EAT and M-EAT (orange line, **Figure S2B(a)**). Second, the adipocytes were separated along the cell contour, which was set by the color threshold (red line, **Figure S2B(b)**). The vessels that were misidentified for adipocytes were manually removed. Third, the adipocytes located on the ROI border line were automatically eliminated. The adipocytes located entirely in the ROI were identified (**Figure S2B(c)**). Finally, cross-sectional area, total number of adipocytes, and mean adipocyte diameter were automatically calculated within the ROI (**Figure S2(d)**) (C-EAT: mean area 415,799 ± 649,239 μm^2^, mean number of cells 163 ± 238 cells, mean adipocyte diameter 57 ± 8.4 μm, M-EAT: mean area 96,851 ± 76,418 μm^2^, mean number of cells 65 ± 53 cells, and mean adipocyte diameter 44 ± 5.9 μm). These visual assessments were validated in a subset of samples using semi-automated digital processing (Keyence).

In the following paragraphs, we defined ‘EAT fibrosis in M-EAT’ as the ‘EAT fibrosis’.

**Immunohistochemistry**

LAA specimens were fixed in 4% paraformaldehyde, embedded in paraffin, and cut into 5-µm sections that were labelled with primary antibodies against cluster of differentiation (CD)3, CD68, α-smooth muscle actin (SMA) (all from Abcam, Cambridge, UK), and the appropriate biotin-conjugated secondary antibody (ABC reagent; Vector Laboratories, Burlingame, CA, USA or DAKO EnVision™+ System, Peroxidase; DakoCytomation, Glostrup, Denmark). Images were acquired and digitized on a BZ-9000 Biolevo epifluorescence microscope with an attached digital camera (Keyence).

**Quantitative real-time polymerase chain reaction (qRT-PCR)**

Transcriptional expression level was determined by qRT-PCR as previously described ^4^. Total RNA was extracted from atrial myocardium using the ISOGEN II (Nippongene, Toyama, Japan), and cDNA was synthesized from total RNA using the Transcriptor 1_st_ Strand cDNA Synthesis Kit (Roche Diagnostics, Mannheim, Germany), following the manufacturers’ protocols. The amplification was performed with the Universal Probe Library on a LightCycler 480 probe master instrument (Roche Diagnostics, Rotkreuz, Switzerland) using the TaqMan method. Primer sequences and conditions are listed in **Table S1**.

**Gene expression microarrays extracted from adipocytes**

Laser micro-dissection was performed using the PALM MicroBeam micro-dissection apparatus (Carl Zeiss, Oberkochen, Germany) (**Figure S4**). The total RNA was extracted from sections using the Qiagen AllPrep DNA/RNA FFPE kit (Qiagen, Dusseldorf, Germany). RNA samples were quantified by an ND-1000 spectrophotometer (NanoDrop Technologies, Wilmington, DE, USA) and the quality was confirmed with a Tapestation (Agilent technologies, Santa Clara, CA, USA). The cRNA was amplified, labeled with total RNA using GeneChip® WT Pico Kit, and hybridized to Thermo Fisher Scientific Clariom™ D Assay, human according to the manufacturer's instructions. All hybridized microarrays were scanned by an Affymetrix scanner. Relative hybridization intensities and background hybridization values were calculated using Affymetrix Expression Console™. The raw signal intensities of all samples were normalized by quantile algorithm with Affymetrix® Power Tool version 1.15.0 software.

Gene expression in C-EAT and M-EAT was measured and their ratio was designated as C-EAT/M-EAT ratio. Three typical gene groups, representing inflammation, fibrosis, and adipogenesis were summarized in the figure.

Microarray data sets have been submitted to the GEO-NCBI with the accession number: GSE154436.

**Culture of EAT**

EAT obtained from one LAA sample was immediately placed on a dish with preadipocyte differentiation medium (PromoCell, Heidelberg, Germany), and cut into 40 pieces, approximately 1 mg per pieces. They were divided into 4 groups (n=10 for each group), and phosphate-buffered saline (PBS) (as a control), including interleukin (IL)-6 (25 ng/ml), transforming growth factor (TGF)-β1 (1 ng/ml), and tumor necrosis factor (TNF)-α (5 ng/ml) were added in each group. After culturing for 24 hours in an incubator at 37°C under 5%CO_2_/95% air atmosphere, they were used for quantifying the transcriptional expression levels.

**Protein concentration of cytokines/chemokines in EAT and total collagen in the atrial myocardium**

The protein content of pro-inflammatory and pro-fibrotic cytokines/chemokines in EAT in 59 out of 76 samples, and the protein content of collagen in the atrial myocardium in 66 out of 76 samples were analyzed. They were evaluated which had sufficient quality for analysis, as previously described ^3^. EAT was homogenized in 250 μl of ice-cold homogenization buffer using a Bio-Plex cell lysis kit (Bio-Rad Laboratories, Milan, Italy). The homogenate was centrifuged at 3,000 × g for 15 min at 4 °C. The ‘fat cake’ was discarded and the homogenate centrifuged again at 14,000 × g for 20 min at 4 °C. The supernatant was stored in aliquots at −70 °C. The protein content of pro-inflammatory and pro-fibrotic cytokines/chemokines in EAT was determined using magnetic bead multiplex immunoassays (Bio-Plex, Bio-Rad Laboratories). Luminex multiplex panel technology was used for simultaneous measurement of a panel of 27 analytes, including pro-inflammatory and pro-fibrotic cytokines/chemokines. In brief, 50 μl of samples and standards were added in duplicate to a 96-multiwell plate containing analyte beads. After incubation for 30 min at room temperature and washing, an antibody-biotin reporter was added and incubated for 10 min with streptavidin-phycoerythrin. The concentrations of the cytokines/chemokines were determined using a Bio-Plex array reader (Luminex, Austin, TX, USA). Bio-Plex Manager software optimized the standard curves automatically and returned the data as median fluorescence intensity (MFI) and concentration (pg/ml). The protein content of pro-inflammatory and pro-fibrotic cytokines/chemokines in EAT was normalized to the content of total protein (1 mg/ml). The total protein concentration of each sample was estimated using the Bradford protein quantification assay.

The protein content of matrix metalloproteinase (MMP)2 and MMP9 in EAT homogenates was measured by ELISA according to manufacturer instructions (Abcam, Cambridge, UK). Expression of Angptl2 and Angptl4 in EAT homogenates was measured by ELISA according to manufacturer instructions (IBL, Fujioka, Japan).

The protein content of total collagen in the atrial myocardium was determined using a commercially available QuickZyme total collagen assay (QuickZyme Bioscience, Leiden, Netherlands) and normalized to tissue weight (1 mg).

**Electron microscopy**

To visualize the sectional ultrastructure of EAT and the atrial myocardium, the remaining paraffin blocks of LAA specimens were used for scanning electron microscopy (SEM). They were deparaffinized with xylene, hydrated, and fixed again in 2.0% paraformaldehyde and 2.5% glutaraldehide, and immersed in 2N NaOH at 37 °C for 3 h to expose myofibrils. Specimens were placed in 1% osmium tetroxide, 1% tannic acid and 1% osmium tetroxide for 1-h each, dehydrated in ethanol of ascending concentrations and then dried by the tert-butylalchol freeze-drying method. The specimens were coated with gold, and examined at 15 kV on a scanning electron microscope (S-4800; Hitachi High-Technologies, Tokyo, Japan). Specimens were post-fixed for 30 min at room temperature in 1% OSO4, dehydrated in a graded series of ethanol, and embedded in epoxy resin. Semi-thin sections (1.0-μm thick) were stained with 1% toluidine blue for light microscopy. Then, ultra-thin sections (80–100-nm thick) were stained with uranyl acetate and lead citrate and examined using transmission electron microscopy (TEM) using a JEM-1200EXII system (JEOL, Tokyo, Japan).

**EAT volume quantification**

Multidetector CT examinations were performed on 320-channel CT scanners (Aquilion ONE, ONE Genesis; Canon Medical Systems Corporation, Tochigi, Japan). The following scan parameters were used: 120 kVp; 200–300 mA; 40-mm beam collimation; pitch of 0.813; 0.5-s rotation; 1.0-mm slice thickness at an interval of 1.0 mm. Non-contrast whole-body CT images were obtained with the patients in a supine position. Images were displayed with a matrix of 512 by 512 pixels (field of view; median 365 mm [interquartile range, 350–387.5 mm], pixel size; median 0.71 mm [0.68–0.76 mm]) and captured at window settings that allowed viewing of the mediastinum (window level, 30 Hounsfield Unit (HU); window width, 300 HU). The EAT volume was quantified semi-automatically by a high-speed three-dimensional image-analysis system (Synapse Vincent; Fuji Photo Film, Tokyo, Japan) on CT images (**Figure S5A**). The pericardium was traced manually from the right pulmonary artery to the diaphragm, to determine the region of interest (ROI). Within the ROI, adipose tissue was defined as pixels within a window of −195 to −45 HU as previously described ^3^. Overall, only pixels with a HU equivalent to adipose tissue within the pericardial sac were considered as EAT.

**CT imaging to determine percent (%) change in EAT fat attenuation**

To evaluate the EAT fat attenuation using CT images, a high-speed three-dimensional image-analysis system (Synapse Vincent; Fuji Photo Film) was used. Two cardiologists (Y.I. and I.A.) blinded to all patient information reviewed the CT scans. EAT analysis was performed on axial images above the origin of the left coronary artery and below the pulmonary artery bifurcation where the cross-sectional EAT area was at a maximum (dotted yellow line in **Figure S5B(a)(b)(c)**). First, EAT was defined within a window of −195 to −45 HU ^3^. Second, the two closest LAA points to both the aorta and pulmonary artery were determined and a line was drawn between them (**Figure S5B(d)**). Another line was drawn perpendicular to LAA from the midpoint of the abovementioned line. Then, a tentative starting point was set at the EAT edge close to LAA. From the tentative starting point, bi-lateral lines parallel to LAA surface were drawn. The 9 × 9 pixels were created from this line to distal EAT (**Figure S5B(e)**). The mean CT fat attenuation for each pixel was calculated automatically (**Figure S5B(f)**). The 9 × 9 pixels were defined as ROI in this study because they were at a maximum range without interference from other structures, such as aorta and pulmonary artery, in all subjects. Finally, the true starting point was adjusted to the most proximal nine pixels that showed a value of ≤ -45 HU. The 3D heat map was created for each patient within the 9 × 9 pixels (ROI) using Image J (National Institutes of Health, Bethesda, Maryland, USA) (**Figure S5C(a)**) ^5^. The curve was constructed to follow the mean EAT fat attenuation of each pixel line from LAA toward the center of EAT within the ROI (**Figure S5C(b)**). Following the previous study ^6^, %change in EAT fat attenuation from the maximum CT fat attenuation (proximal side from LAA in EAT) to minimum CT fat attenuation (distal side) was calculated as follows: %change in EAT fat attenuation = 100x(maximum CT fat attenuation – minimum CT fat attenuation)/maximum CT fat attenuation (**Figure S5C(b)**).

**Supplemental Figures**

**Supplemental Figure S1. Analysis of LAA samples.**

LAA sections were prepared for histological and biochemical study. 1) The samples fixed in 4% paraformaldehyde were embedded in paraffin and stained with hematoxylin and eosin, Masson’s trichrome, and immunochemistry. They were also used for the micro-dissection and microarray. 2) The samples fixed in mixture of 2.0% paraformaldehyde and 2.5% glutaraldehyde were examined by electron microscopy. 3) The samples stored in -80℃ freezer were assayed to quantify the proteins and gene expressions. LAA, left atrial appendage.

**Supplemental Figure S2. Histological quantification of LAA sections.**

**A:** Representative EAT fibrosis and myocardial fibrosis measurements. Each area was identified by manual delineation (C-EAT: orange, M-EAT: dark green). The fibrosis area was detected by a color-extraction method (EAT fibrosis in C-EAT: yellow, EAT fibrosis in M-EAT: green, Myocardial fibrosis: water blue). **B:** Representative adipocyte diameter measurement. **(a)** The ROI was identified by manual delineation. **(b)** The adipocytes were separated along the cell contour which was set by the color threshold. **(c)** The diameter of adipocytes was calculated automatically eliminating the adipocytes which straddled the border of ROI. **(d)** Representative adipocytes in C-EAT and M-EAT were shown. MT, Masson’s trichrome; ROI, region of interest.

**Supplemental Figure S3. Relationship between myocardial fibrosis and EAT fibrosis.**

**A:** Representative cases of mildly and severely fibrotic remodeling of EAT. **B:** Myocardial fibrosis was positively correlated with EAT fibrosis. EAT fibrosis was greater in the persistent atrial fibrillation (PeAF) group than in the paroxysmal atrial fibrillation (PAF) group. PAF, paroxysmal atrial fibrillation; PeAF, persistent atrial fibrillation.

**Supplemental Figure S4. Micro-dissection of C-EAT and M-EAT for microarray analysis.**

Laser micro-dissection was performed from each adipose tissue in sections (C-EAT: blue, M-EAT: red), and used for the microarray analysis. C, C-EAT; M, M-EAT.

**Supplemental Figure S5. Quantification of epicardial adipose tissue (EAT) volume and %change in EAT fat attenuation on CT images.**

**A:** Representative measurement image and three-dimensional image of EAT on a Synapse Vincent system. **B:** Representative measurement of EAT fat attenuation on CT images. **(a)(b)(c)** The analyzed CT images with axial plane including the maximum depiction of EAT area above the origin of left coronary artery and below the pulmonary artery bifurcation. **(d)(e)(f)** Determination of ROI. The starting point was determined as the point of intersection of the EAT edge close to LAA and the perpendicular line from the midpoint between each closest point of LAA to aorta and LAA to pulmonary artery. As ROI, 9 × 9 pixels was drawn from the edge line centered on the starting point to the center of EAT. The yellow number is the EAT fat attenuation satisfying the window of -195 to -45 HU. **C: (a)** Representative three-dimensional heat map of EAT fat attenuation within ROI. **(b)** The curve derived from the EAT fat attenuation from LAA toward center of EAT. The %change in EAT fat attenuation was calculated as follow: %change in EAT fat attenuation = 100x(maximum CT fat attenuation - minimum CT fat attenuation)/maximum CT fat attenuation. LAA, left atrial appendage; LCA, left coronary artery; LA, left atrium; Ao, aorta; PA, pulmonary artery; ROI, region of interest.

**References**

**1.** Gage BF, Waterman AD, Shannon W, Boechler M, Rich MW, Radford MJ. Validation of clinical classification schemes for predicting stroke: results from the National Registry of Atrial Fibrillation. JAMA Jun 13 2001;285:2864-2870.

**2.** Lip GY, Nieuwlaat R, Pisters R, Lane DA, Crijns HJ. Refining clinical risk stratification for predicting stroke and thromboembolism in atrial fibrillation using a novel risk factor-based approach: the euro heart survey on atrial fibrillation. Chest Feb 2010;137:263-272.

**3.** Abe I, Teshima Y, Kondo H, et al. Association of fibrotic remodeling and cytokines/chemokines content in epicardial adipose tissue with atrial myocardial fibrosis in patients with atrial fibrillation. Heart Rhythm Nov 2018;15:1717-1727.

**4.** Abe I, Terabayashi T, Hanada K, et al. Disruption of Actin Dynamics Regulated by Rho Effector mDia1 Attenuates Pressure Overload-Induced Cardiac Hypertrophic Responses and Exacerbates Dysfunction. Cardiovasc Res Jul 9 2020.

**5.** Schneider CA, Rasband WS, Eliceiri KW. NIH Image to ImageJ: 25 years of image analysis. Nat Methods Jul 2012;9:671-675.

**6.** Antonopoulos AS, Sanna F, Sabharwal N, et al. Detecting human coronary inflammation by imaging perivascular fat. Sci Transl Med Jul 12 2017;9.

**Supplemental Tables**

**Supplemental table S1: The Primer sequences used for qRT-PCR.**

| Human |  |  |  |  |  |
| --- | --- | --- | --- | --- | --- |
| Gene | Forward Primer | | Reverse Primer | | Universal  Probe no. |
| FABP4 | 5'-CCTTTAAAAATACTGAGATTTCCTTCA-3' | | 5'-GGACACCCCCATCTAAGGTT-3' | | #72 |
| CEBPA | 5'-GGAGCTGAGATCCCGACA-3' | | 5'-TTCTAAGGACAGGCGTGGAG-3' | | #28 |
| PPAR-γ | 5'-GACAGGAAAGACAACAGACAAATC-3' | | 5'-GGGGTGATGTGTTTGAACTTG-3' | | #7 |
| ADIPOQ | 5'-AGAGATGGCACCCCTGGT-3' | | 5'-CCGGTTTCACCGATGTCT-3' | | #85 |
| PLIN1 | 5'-TGAACATTAAAGGGAAGAAGTTGAA-3' | | 5'-TTCTCCTGCTCAGGGAGGT-3' | | #42 |
| SLC2A4 | 5'-GGCATGGGTTTCCAGTATGT-3' | | 5'-GCCTCGAGTTTCAGGTACTCTT | | #3 |
| AGPAT2 | 5'-AGGGTACTCGCAACGACAAT-3' | | 5'-GTACACCACGGGGACGAT-3' | | #62 |
| LEP | 5'-TTGTCACCAGGATCAATGACA-3' | | 5'-GTCCAAACCGGTGACTTTCT-3' | | #25 |

**Supplemental Table S2: Top 30 genes upregulated and downregulated in C-EAT compared to M-EAT**

| **Top 30 genes upregulated in C-EAT compared to M-EAT** | | |
| --- | --- | --- |
| **Gene** | **Gene Accession No.** | **C-EAT / M-EAT** |
| IL7R | NM_002185 | 32.3 |
| JCHAIN | NM_144646 | 28.7 |
| TRIM59 | NM_173084 | 24.4 |
| IGLC3 | OTTHUMT00000321821 | 22.4 |
| KLRB1 | NM_002258 | 20.0 |
| RGS1 | NM_002922 | 19.5 |
| IGLC7 | OTTHUMT00000320966 | 14.6 |
| FDCSP | NM_152997 | 13.7 |
| IGHA1 | OTTHUMT00000326459 | 13.1 |
| FLG | NM_002016 | 11.3 |
| SPIC | NM_152323 | 10.7 |
| IGLC6 | OTTHUMT00000321634 | 9.3 |
| HK1 | NM_000188 | 9.0 |
| IGLL5 | NM_001178126 | 8.7 |
| RFC4 | NM_002916 | 8.4 |
| PRKAG1 | NM_001206709 | 7.6 |
| IGHG3 | BC089421 | 7.4 |
| AFG3L2 | NM_006796 | 6.8 |
| INTS12 | NM_001142471 | 6.5 |
| ZFYVE21 | NM_001198953 | 6.5 |
| MAN1A1 | NM_005907 | 6.1 |
| MPHOSPH6 | NM_005792 | 6.1 |
| FAM58A | NM_001130997 | 5.9 |
| POPDC3 | NM_022361 | 5.7 |
| TSPAN6 | NM_001278740 | 5.7 |
| CCDC109B | NM_017918 | 5.6 |
| PUS7 | NM_019042 | 5.5 |
| PRG4 | NM_001127708 | 5.4 |
| CCDC14 | NM_001308317 | 5.3 |
| CCNE2 | NM_057749 | 5.2 |

| **Top 30 genes downregulated in C-EAT compared to M-EAT** | | |
| --- | --- | --- |
| **Gene** | **Gene Accession No.** | **C-EAT / M-EAT** |
| CDC14B | NM_001077181 | 0.059 |
| SMAD6 | NM_005585 | 0.065 |
| SCRG1 | NM_007281 | 0.107 |
| IFT88 | NM_006531 | 0.111 |
| VCAN | NM_001126336 | 0.119 |
| LRRC37A | NM_014834 | 0.121 |
| NPY6R | NR_002713 | 0.125 |
| PTCH1 | NM_000264 | 0.140 |
| NPPA | NM_006172 | 0.141 |
| ZEB1 | NM_001128128 | 0.142 |
| PSMA6 | NM_001282232 | 0.144 |
| MAF | NM_001031804 | 0.151 |
| PIK3R1 | NM_001242466 | 0.155 |
| ZNF638 | NM_001014972 | 0.158 |
| CEP70 | NM_001288964 | 0.159 |
| HIBCH | NM_014362 | 0.164 |
| MTFR1L | NM_001099625 | 0.170 |
| UROS | NM_000375 | 0.172 |
| CDS2 | NM_003818 | 0.172 |
| ST7L | NM_001308264 | 0.173 |
| EIF3M | NM_001307929 | 0.174 |
| PRPSAP1 | NM_002766 | 0.180 |
| FAN1 | NM_001146094 | 0.188 |
| ABCC9 | NM_005691 | 0.192 |
| CTSB | NM_001908 | 0.192 |
| ABI3BP | NM_015429 | 0.195 |
| LCLAT1 | NM_001002257 | 0.195 |
| CORO1C | NM_001105237 | 0.196 |
| NUCB2 | NM_005013 | 0.196 |
| OGN | NM_014057 | 0.199 |

**Supplemental Table S3: Relationship between C/M diameter ratio and cytokines/chemokines in EAT**

| Correlation between C/M diameter ratio and cytokines/chemokines in EAT | | | |
| --- | --- | --- | --- |
|  | Univariable analysis |  |  |
| Variable | Pearson r | r^2^ | p value |
| IL-1b* | 0.304 | 0.092 | 0.030 |
| IL-1ra | 0.172 | 0.030 | 0.228 |
| IL-2† | 0.441 | 0.194 | 0.009 |
| IL-4* | 0.229 | 0.052 | 0.106 |
| IL-5 | 0.025 | 0.001 | 0.887 |
| IL-6* | 0.215 | 0.046 | 0.130 |
| IL-7† | 0.467 | 0.218 | <0.001 |
| IL-8 | 0.156 | 0.024 | 0.275 |
| IL-9* | 0.357 | 0.127 | 0.010 |
| IL-10* | 0.400 | 0.160 | 0.016 |
| IL-12† | 0.472 | 0.223 | 0.002 |
| IL-13† | 0.520 | 0.270 | <0.001 |
| IL-15 | 0.017 | <0.001 | 0.910 |
| IL-17* | 0.280 | 0.078 | 0.049 |
| Eotaxin† | 0.549 | 0.301 | <0.001 |
| basic FGF | 0.165 | 0.027 | 0.249 |
| G-CSF* | 0.357 | 0.127 | 0.012 |
| GM-CSF* | 0.264 | 0.070 | 0.087 |
| IFN-γ† | 0.416 | 0.173 | 0.002 |
| IP-10* | 0.301 | 0.091 | 0.032 |
| MCP-1* | 0.321 | 0.103 | 0.021 |
| MIP-1α | 0.191 | 0.036 | 0.179 |
| MIP-1β* | 0.270 | 0.073 | 0.056 |
| PDGF-BB† | 0.535 | 0.286 | <0.001 |
| RANTES† | 0.480 | 0.230 | <0.001 |
| TNFα† | 0.579 | 0.335 | <0.001 |
| VEGF* | 0.260 | 0.068 | 0.081 |
| MMP2† | 0.419 | 0.176 | 0.002 |
| MMP9* | 0.212 | 0.045 | 0.135 |
| ANGPTL2* | 0.292 | 0.085 | 0.038 |
| ANGPTL4 | 0.105 | 0.011 | 0.463 |

IL = interleukin; FGF =fibroblast growth factor; G-CSF = granulocyte-colony stimulating factor;　GM-CSF = granulocyte macrophage-colony stimulating factor; IFN = interferon; IP = interferon-induced protein; MCP = monocyte chemoattractant protein; MIP = macrophage inflammatory protein; PDGF = platelet derived growth factor-BB; TNF = tumor necrosis factor; VEGF = vascular endothelial growth factor; MMP = matrix metalloproteinase; ANGPTL = angiopoietin-like protein.

*0.2<|r|≤0.4

†0.4<|r|≤0.7
